# The Effects of Isolated Fractions of *Mesobuthus eupeus* Scorpion Venom on Humoral Immune Response

**Published:** 2017-12-30

**Authors:** Mohammad Khosravi, Mansour Mayahi, Farnoosh Kaviani, Mohammad Nemati

**Affiliations:** 1 Department of Pathobiology, Faculty of Veterinary Medicine, Shahid Chamran University of Ahvaz, Ahvaz, Iran; 2 Department of Clinical Sciences, Faculty of Veterinary Medicine, Shahid Chamran University of Ahvaz, Ahvaz, Iran; 3 Student of Veterinary Medicine, Faculty of Veterinary Medicine, Shahid Chamran University of Ahvaz, Ahvaz, Iran; 4 Razi Reference Laboratory of Scorpion Research, Razi Vaccine and Serum Research Institute, Karaj, Iran

**Keywords:** Venom, *Mesobuthus eupeus*, Immune response

## Abstract

**Background:**

Many elements such as immunosuppressive, chemotactic and anti-inflammatory peptide that could effect on human and animals physiologic system were determined in venom. This study evaluated the use of *Mesobuthus eupeus* scorpion venom fractions as an immunomodulator.

**Methods:**

The venom fractions collected from Khuzestan Province in South West of Iran were purified by ion exchange chromatography. Elution of the bounded elements was done by using a linear gradient of sodium chloride (0.1, 0.25, 0.5, 0.75, 1, 1.25, 1.5 and 2 molar). The fractions were analyzed by Bradford spectrophotometric and SDS-PAGE method. After treatments of chicken with venom fractions and sheep red blood cell (SRBC), direct haemagglutination test in microtiter plate was used for the determination of the chicken SRBC antibody titer.

**Results:**

The fraction released by NaCl 1.25M had the highest protein concentration. The highest and lowest antibody titer was determined at the fifth (NaCl 0.75 molar) and seventh fraction (NaCl 1.25 molar), respectively.

**Conclusion:**

Different protein profile of isolated fractions, were associated with various effect on immune response. Both enhancing and suppressing of the chicken humoral immune response to SRBC were observed after *M. eupeus* faction’s venom treatment. It is due to biological functions of venom components. Purification of these elements would provide the new agents for immune responses manipulation.

## Introduction

Scorpion venom contains biological compounds as short-chain peptides, bioactive substances such as enzymes, nucleotides, lipids, mucoprotein, mucopolysaccharides, biogenic amines, neuroactive peptides, protease inhibitors, phospholipase, hyaluronidase (1) and other unknown compounds, which could affect on the physiologic system of vertebrate and invertebrate organisms (2). Only 400 peptides out of the expected 100000 peptides of venom have toxic effects on human and animals (3). Envenomated organism produces various mediators, which contains both pro- and anti-inflammatory cytokines (4). Scorpion venom could induce local and systemic inflammatory responses. The local effects can lead to the activation of vascular endothelium, increase the vascular permeability and leukocytes migration to the affected tissues. The systemic inflammation persuades the acute phase response (5). The consequence of this inflammatory reaction is undergoing by a variety of factors such as duration of the stimulus and the balance between the inflammatory and anti-inflammatory mediators (4).

Different methods, used for fractionation, purification, analysis of the structure and characterization of toxins have made it possible to clarify the components of venoms. Identification of toxins or others biologically important peptide were the outcome of these efforts (6). Many elements such as immunosuppressive (7) chemotactic (8, 9) and anti-inflammatory peptides were determined in venom and are as potential therapeutic agents (10). Scorpion’s venom has been used in medicine for relation of pain, osteoporosis, neurological diseases, homeostasis and rheology, cancer and autoimmune disease (11, 12).

Scorpions were divided into 13 families and about 1400 species and subspecies (13). The medical importance scorpion species, belonging to the family Buthidae mainly, *Androctonus*, *Buthus*, *Mesobuthus*, *Buthotus*, *Parabuthus*, and *Leirus* (4). The *M. eupeus* venom could affect on cytokine releases with both pro and anti-inflammatory properties (14, 15). Venom components that affect on immune response could be used as immunomodulatory agents.

We investigated the possibility of using different *M. eupeus* scorpion venom fractions as immunomodulator like elements for elevation or suppression of immune response.

## Materials and Methods

### Venom

*Mesobuthus eupeus* scorpions were collected from Khuzestan Province in South West of Iran (31°19′–32°73′N, 48°41′–49°4′E) milked by electric stimulation. The total protein concentration was measured using the usual Bradford spectrophotometric method with bovine serum albumin (BSA) as standard.

### Chickens

Forty adult Rass 308 chickens were selected and kept in the isolation facility. Feed and water were provided during the experiment. Experimental procedures were according to the guidelines of the animal care.

### Purification of venom fractions

The venom fractions were purified by ion exchange chromatography. Anion-exchange chromatography was performed using diethylaminoethyl cellulose (DEAE-C) column (Sigma, Product Number: D3764) at a flow rate of 1ml/min. The column-stabilizing buffer was 0.05 molar Tris-HCl, pH 8.6. Tow milliliter of *M. eupeus* venom were dissolved in stabilizing buffer and loaded on the column (12 mg/mL). After washing away of the unbound components, elution of the bound elements was done by using a linear gradient of sodium chloride (0.1, 0.25, 0.5, 1, 1.25, 1.5, 1.75 and 2 molar) in 3ml of stabilizing buffer. All the fractions were dialyzed overnight against distilled water, pH 7.2 at 4 °C. The protein concentrations of the collected fractions were measured by Bradford spectrophotometric method (Accu Reader, Serial No: 96501575) with BSA as standard.

### SDS-PAGE

The protein profiles of crude venom, as well as the purified fractions, were analyzed by SDS-PAGE (16), the stacking and resolving gel concentration was 4% and 11%, respectively. Samples were denatured by boiling in loading buffer containing SDS and β-mercaptoethanol prior to loading onto the gel. Proteins were stained with 1% Coomassie blue R 250. Molecular mass standard (Vivantis, product No: PR0602) was run in parallel in order to calculate molecular weights of the proteins.

### Immunomodulatory analysis of the purified fractions

The purified fractions were diluted to 200 μg/ml with pyrogen-free water. The 0.5ml of each fraction injected subcutaneously at breast region of four adult chickens. The sheep red blood cells (SRBC) were prepared by centrifugation of Ethylenediaminetetraacetic acid (EDTA) anticoagulated whole blood, followed by 3 times washing with normal saline. The suspension of fresh 2% SRBC was prepared and the 0.5ml injected in intra-muscularly route. The clinical symptoms were recorded during the test periods. Two ml of the venous blood were collected from the injected chickens 5 and 10 d after the injections. The serum was separated and kept at minus 20 °C until use.

### Hemagglutination test

Direct hemagglutination test in microtiter plate used for the determination of the SRBC antibody titer. Serum samples were serially diluted in a microtiter plate and 1% SRBCs were added. After mixing, the plates were incubated at 37 °C for 45min and examined for hemagglutination. The test repeated 3 times for each samples and antibody titer was determined as the equal of the maximum dilution that exhibiting hemagglutination.

## Results

### Ion exchange chromatography

The total protein concentration of the eluted fractions has been measured and as shown in Table 1, the eluted fraction by NaCl 1.25M had the highest percentage of protein.

**Table 1.** The total protein concentration of the venom fractions which eluted by a linear gradient of sodium chloride

**Elution (Molar)**	**Tris 0.05**	**NaCl 0.1**	**NaCl 0.25**	**NaCl 0.5**	**NaCl 0.75**	**NaCl 1**	**NaCl 1.25**	**NaCl 1.5**	**NaCl 2**
**Protein Concentration (mg/ml)**	4.2	1.27	1.20	1.61	3.95	3.8	4.3	2.05	0.82

### SDS-PAGE

**Fig. 1.**
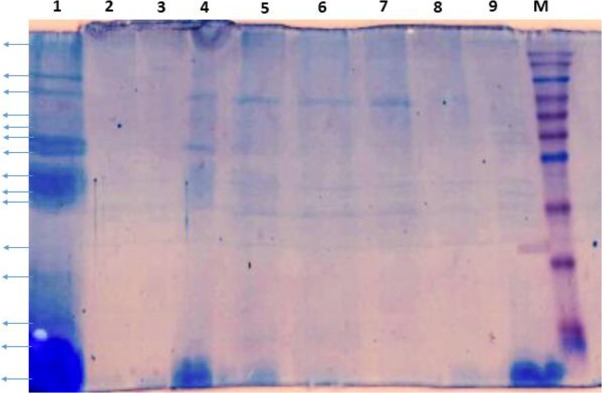
The sodium dodecyl sulfate-polyacrylamide gel electrophoresis analysis of *Mesobuthus eupeus* scorpion venom. The Lane M: Marker proteins (175, 130, 95, 70, 62, 51, 42, 29, 22, 14 and 10, respectively). The lanes 1 to 9 are eluted fractions by 0.05 M Tris-HCl and 2, 1.5, 1.25, 1, 0.75, 0.5, 0.25 and 0.1 M of NaCl, respectively

Venom fractions had a different protein profile (Fig. 1). Proteins of the venom had 15 detectable bands, which were between ≤ 5 and ≥ 175 kDa on 12% polyacrylamide gel electrophoresis.

### Hemagglutination test

The fifth fraction (NaCl 0.75) had the lowest hemagglutination titer, this was less than the control titer. The seventh fraction (NaCl 1.25) had the highest anti SRBC titer (Table 2).

**Table 2.** The hemagglutination titer against sheep red blood cells (SRBC) in chickens which treated with venom fractions or phosphate buffered saline (PBS)

**Fraction (Molar)**	**PBS**	**SRBC**	**1**	**2**	**3**	**4**	**5**	**6**	**7**	**8**	**9**
**5 days titer**	0	10	10	20	30	20	5	30	60	20	10
**10 days titer**	0	20	20	40	40	30	10	40	160	40	20

## Discussion

The isolated fractions of *M. eupeus* venom have immunomodulatory effects. The humoral immune response to the SRBC was a sensitive endpoint to evaluate drug modification of the humoral immunity (17). The cooperation and interaction of antigen presenting cells, T helper and B cells are involved in the production of the anti-SRBC (17). Both inductions and suppression of the humoral immune response to SRBC were observed in treated chickens with venom fractions. The six fractions out of nine fractions increase the anti SRBC titer, from 1.5 to 4 fold. Several proinflammatory mediators, such as leukotrienes, phospholipases A2, prostaglandins, kinins, H2O2, NO production and activation of complement system, were increased in scorpion envenomated peoples (18–20). The fractionated extracts of venom may improve the phagocytic efficacy of the PMN cells and stimulate innate immune response. In addition, venom induces the release of cytokine and activation of endogenous immunological and inflammatory mediators (18). Moreover, scorpion venoms could enhance the release of different inflammatory mediators which cause leukocytosis and raise the cytokines levels such as IL1b, IL6, IL8, IL10, TNFa and NO (21, 22). A mixture of peptide with diverse potential for induction of pro-inflammatory mediator can exist in *M. eupeus* venom. Production of IFN-γ and IL-4 enhance the effect of venom on innate and humoral immune responses (23). Improved humoral response to SRBC was reported for the administration of NNAV (23). The elevation of Th1 and higher proinflammatory response were induced by *Bothrops* venom (24). The venom of *Hemiscorpius lepturus* and *Androctonus crassicauda* scorpion stimulate the monocytes immune response by IL12 production (25, 26).

An inhibitory effect on humoral immune response of chickens to SRBC was observed on one fraction of *M. eupeus* venom. The *M. eupeus* venom was contained effective anti-inflammatory mediators (14). In addition, the other venom has suppressive effects on immune system. Significant inhibition of the immune responses and interfering with the synthesis of immunoglobulin G were observed after viper snakes venom treatment (27, 28). Some of venom derived peptides have immunosuppressant properties and used for the treatment of autoimmune diseases and the organ transplantation (29). In addition, mucopolysaccharides of venom inhibit nitrous oxide and interleukin human chondrocytes (1), hence have anti-inflammatory properties. The *Naja naja atra* venom had anti-inflammatory effects (30). The cobratoxin had anti-inflammatory and inhibitory effect on activation of NF-κB (31).

Both pro- and anti-inflammatory cytokines were produced after scorpion envenomation (22, 32). The IFN-γ, TNF-α, GM-CSF IL-1α, IL-6 and IL-10 levels were increased in envenomed peoples (22, 33). The *B. erythromelas* and *C. d. cascavella* venom have significant immunomodulatory effects. However, *B. erythromelas* enhance a proinflammatory profile and that *C. d. cascavella* venom has anti-inflammatory effects (34). The venom of *T. serrulatus* contains substances with immunomodulatory effects (35).

## Conclusion

Different protein profiles of isolated fractions of *M. eupeus* venom are associated with various effects on the immune response. Both enhancing and suppressing of the chicken humoral immune response to SRBC were observed after *M. eupeus* faction’s venom treatment, this is due to the biological functions of venom components. However, further researches are needed for confirmation of the results, purification of active elements would provide the new agents for immune system modulation.
